# B3GNT7 regulates mucin O-glycosylation to alleviate colonic inflammation

**DOI:** 10.1186/s12876-024-03287-8

**Published:** 2024-06-17

**Authors:** Tian Wang, Han Sun, Minna Zhang, Peng Shen, Yan Li

**Affiliations:** 1 Department of Medical School, Jiangsu Vocational College of Medicine, Yancheng, China; 2 Department of Gastroenterology, Xuzhou Central Hospital, Xuzhou, China; 3 Department of Gastroenterology, The Affiliated Huaian No. 1 People’s Hospital of Nanjing Medical University, Huai’an, China; 4 Department of Internal Medicine, Huai’an Huai’an Hospital, Huai’an, China

**Keywords:** Ulcerative colitis, B3GNT7, Mucin, O-glycosylation

## Abstract

**Background:**

B3GNT7, a glycosyltransferase of significant importance that is highly expressed in intestinal epithelial cells, plays a pivotal role in intestinal physiological processes. This study elucidates novel insights into the potential role and underlying mechanisms of B3GNT7 in ulcerative colitis (UC).

**Methods:**

An experimental colitis model was induced using DSS in mice to investigate B3GNT7 expression in the colon via transcriptomics and immunohistochemistry. Bioinformatics analysis was employed to delineate the biological functions of B3GNT7. Additionally, the correlation between the transcription levels of B3GNT7 in colonic tissues from patients with UC, sourced from the IBDMDB database, and the severity of colonic inflammation was analyzed to elucidate potential mechanisms.

**Results:**

The DSS-induced colitis model was successfully established, and transcriptomic analysis identified a marked downregulation of B3GNT7 expression in the colonic tissues compared to the controls. Functional enrichment analysis indicated B3GNT7’s predominant role in mucin O-glycosylation. Protein interaction analysis revealed that B3GNT7 predominantly interacts with members of the mucin MUC family, including MUC2, MUC3, and MUC6. In patients with UC, B3GNT7 transcription levels were significantly reduced, particularly in those with moderate to severe disease activity. The expression level of B3GNT7 exhibited a negative correlation with the endoscopic severity of UC. Gene set enrichment analysis (GSEA) further demonstrated significant enrichment of B3GNT7 in the mucin O-glycosylation synthesis pathway.

**Conclusion:**

The downregulation of B3GNT7 expression in the colonic tissues of UC patients may contribute to the compromised mucin barrier function and the exacerbation of colitis.

## Background

Ulcerative colitis (UC), characterized as a chronic, nonspecific inflammatory bowel disorder, has emerged as a significant global public health concern, with its etiological mechanisms remaining incompletely elucidated [1, 2]. As advancements in the comprehension of colitis pathogenesis mechanisms have been achieved in recent years, there has been a burgeoning interest among researchers in the role of B3GNT7 in intestinal epithelial homeostasis [3, 4].

B3GNT7 encodes an enzyme that is critically involved in glycosylation modifications, a process integral to cellular function [5]. Emerging evidence indicates that B3GNT7 is pivotal in modulating the intestinal mucosal barrier, a key determinant in the onset and progression of colitis [3]. By governing the glycosylation of adhesive molecules, such as mucins, B3GNT7 is instrumental in sustaining intestinal homeostasis [3].

As a pivotal enzyme, B3GNT7 is integral to numerous biological processes, and unraveling its significance may yield substantial insights into the complex pathophysiology of UC [4]. Confirming B3GNT7 as a definitive biomarker for UC could significantly enhance our understanding of its pathogenesis and might pave the way for innovative diagnostic and therapeutic strategies. Such a breakthrough could herald new avenues in the management of UC, potentially leading to improved patient outcomes. This study is designed to investigate the specific functions of B3GNT7 in UC and to evaluate its potential as a molecular biomarker.

## Methods

### Animals

C57BL/10J mice (6 to 8 weeks of age; weighing 18–20 g; specific pathogen-free grade) were purchased from the Model Animal Research Center of Nanjing University. All mice were reared at the experimental animal center of the Affiliated Huaian No.1 People’s Hospital of Nanjing Medical University. Throughout the acclimatization and study periods, the mice were maintained in a 12-hour light/12-hour dark cycle. They had ad libitum access to food and water. The mice were group-housed in standard cages. DSS (36–50 kDa) was purchased from MP Biomedicals LLC and dissolved in distilled water. Experimental colitis was induced as previously described with minor changes [6]. For the different groups, the mice were administered 2.5% (w/v) DSS in their drinking water for 7 days. The mice were anesthetized by i.p. injection of pentobarbital sodium, and their colon were harvested for further experiments and then quickly sacrificed by dislocating the neck. The colons were measured for length, and the tissues were examined for gross macroscopic appearance and stool consistency. The distal colon segment was placed in 10% neutral buffered formalin for 24 h, embedded in paraffin, and sectioned into 4 μm thickness. Immunohistochemical methods were used to detect the expression of B3GNT7 protein in the tissue sections. The antibody was purchased from Abcam (AB190217), antibody dilution ratio 1:100. The ethics of this study were reviewed and approved by the Ethics Committee of The Affiliated Huaian No. 1 People’s Hospital of Nanjing Medical University.

### Transcriptome analysis

Total RNA was extracted from inflammatory colonic tissue. For sequencing, a 1 cm section of colon tissue was sampled from a site approximately 2 cm from the anus, regardless of whether there was visible inflammation. Tissue samples with the minimum and maximum histological scores were removed. Then, colon samples from four randomly chosen animals in each group were used for sequencing. The methods for amplifying and sequencing followed those previously published [6]. Briefly, 2 µg RNA per sample was used for sequencing on the Illumina Hiseq 4000 platform. Differential expression analysis was performed using the DESeq R package according to the manufacturer’s protocol. To explore the potential function of the differentially expressed genes (DEGs), the GOseq R package and KOBAS software were used to test the enrichment of DEGs in Gene Ontology (GO) functional annotations and Kyoto Encyclopedia of Genes and Genomes (KEGG) pathways [6, 7]. The RNA-seq data was deposited in the Gene Expression Omnibus (GEO) database (http://www.ncbi.nlm.nih.gov/geo/) under the accession number GSE244377. DEGs were screened by GO and KEGG enrichment analysis, as well as protein-protein interaction (PPI) analysis. The database STRING (version 11.5, http://cn.string-db.org/) and software Cytoscape (v 3.9.1) were utilized to construct hub gene networks.

### IBDMDB Database

The IBDMDB database [8] is a component of the integrative Human Microbiome Project (iHMP). In this study, 23 UC patients and 20 non-IBD patients were selected as controls using the IBDMDB database. These subjects had complete clinical data and samples of intestinal mucosa from rectal biopsies. The transcriptome sequencing data of each sample was re-analyzed using DESEQ2 after screening with the IBDMDB database, allowing for the identification of differentially expressed genes through the calculation of their fold expression changes and P values. To further analyze the differential genes, GO classification enrichment analysis and protein interaction network construction were performed using the online analysis tool Metascape (https://Metascape.org) website. Based on the median expression of the B3GNT7 in colonic tissue samples, the UC patients were divided into two groups: a high-expression group and a low-expression group. GSEA 4.1.0 software was used to perform the analysis with default parameters, including setting the number of permutations to 1000.

### Statistical analysis

Differences were analyzed using the t-test in Graphpad Prism 8.0 software. Results are shown as the mean ± standard error of the mean. *P* < 0.05 was considered statistically significant.

## Results

### DSS induced colitis model in mice

**Fig. 1:**
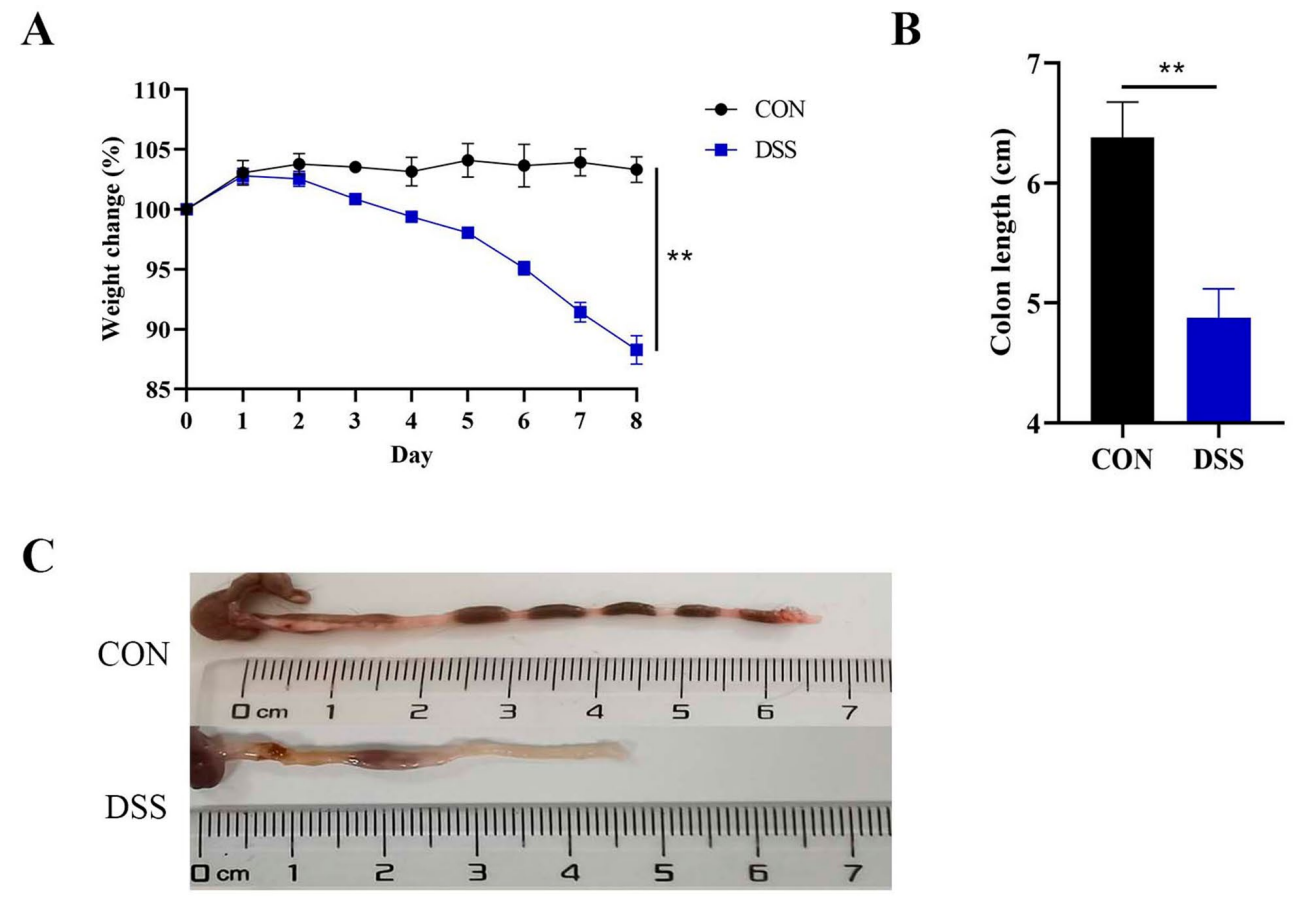
DSS-induced colitis model in mice. **A**. Changes in body weight of colitis mice. **B**. Quantification of colon length. **C**. A significant reduction in colon length were observed in the DSS group when compared to CON group

To establish an experimental colitis model in mice, we induced colitis by providing the mice with 2.5% DSS in their drinking water. On the 8th day post-induction, we conducted a comparative analysis of the alterations in body weight and colon length between the DSS-induced group and the control group (CON). Evaluation of these parameters enabled us to gauge the severity of colitis and discern the effects of DSS-induced inflammation on the mice (Fig. 1). As anticipated, the mice in the DSS group demonstrated a significant decline in body weight, indicative of the detrimental impact of colitis on their overall well-being (*P* = 0.005). Additionally, a notable decrease in colon length was observed in the DSS group relative to the CON group (*P* = 0.004). These results suggest that the provision of 2.5% DSS effectively induced colitis in the mouse model, manifesting in characteristic symptoms such as weight loss and reduced colon length.

### B3GNT7 expression decreased in inflammatory colonic tissue

**Fig. 2:**
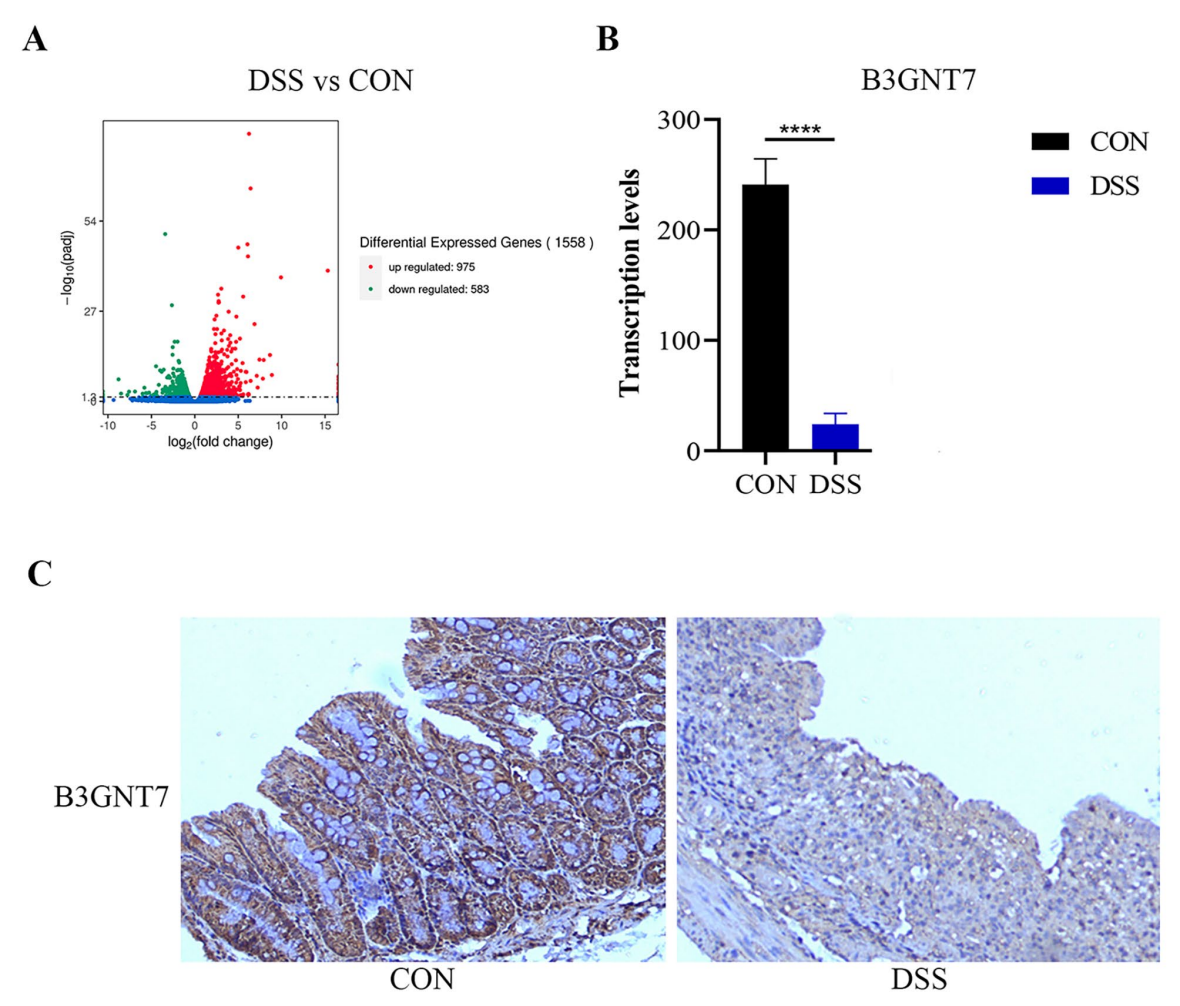
Expression of B3GNT7 in intestinal inflammation. **(A)** Transcriptome sequencing analysis comparing the intestinal tissue transcriptome of mice from the healthy control CON group and the Colitis DSS group, revealing 1558 differentially expressed genes. **(B)** Significant down-regulation of colonic B3GNT7 expression in the DSS group (*P* < 0.0001). **(C)** IHC detection of B3GNT7 protein expression, showing strong positivity in the CON group and weak positivity in the DSS group

Colon tissue specimens were procured for transcriptomic sequencing analysis via RNA-seq. The analysis revealed the existence of 1558 genes exhibiting differential expression between the CON group and the DSS-induced group. At the transcriptional level, a marked decrease in B3GNT7 expression was identified in the DSS group relative to the CON group, with a p-value of less than 0.0001. At the protein level, immunohistochemical evaluation of B3GNT7 protein expression demonstrated that the colonic mucosa in the DSS group exhibited weakly positive expression of B3GNT7, in contrast to the strongly positive expression observed in the CON group (Fig. 2).

### Functional enrichment analysis of B3GNT7

**Fig. 3:**
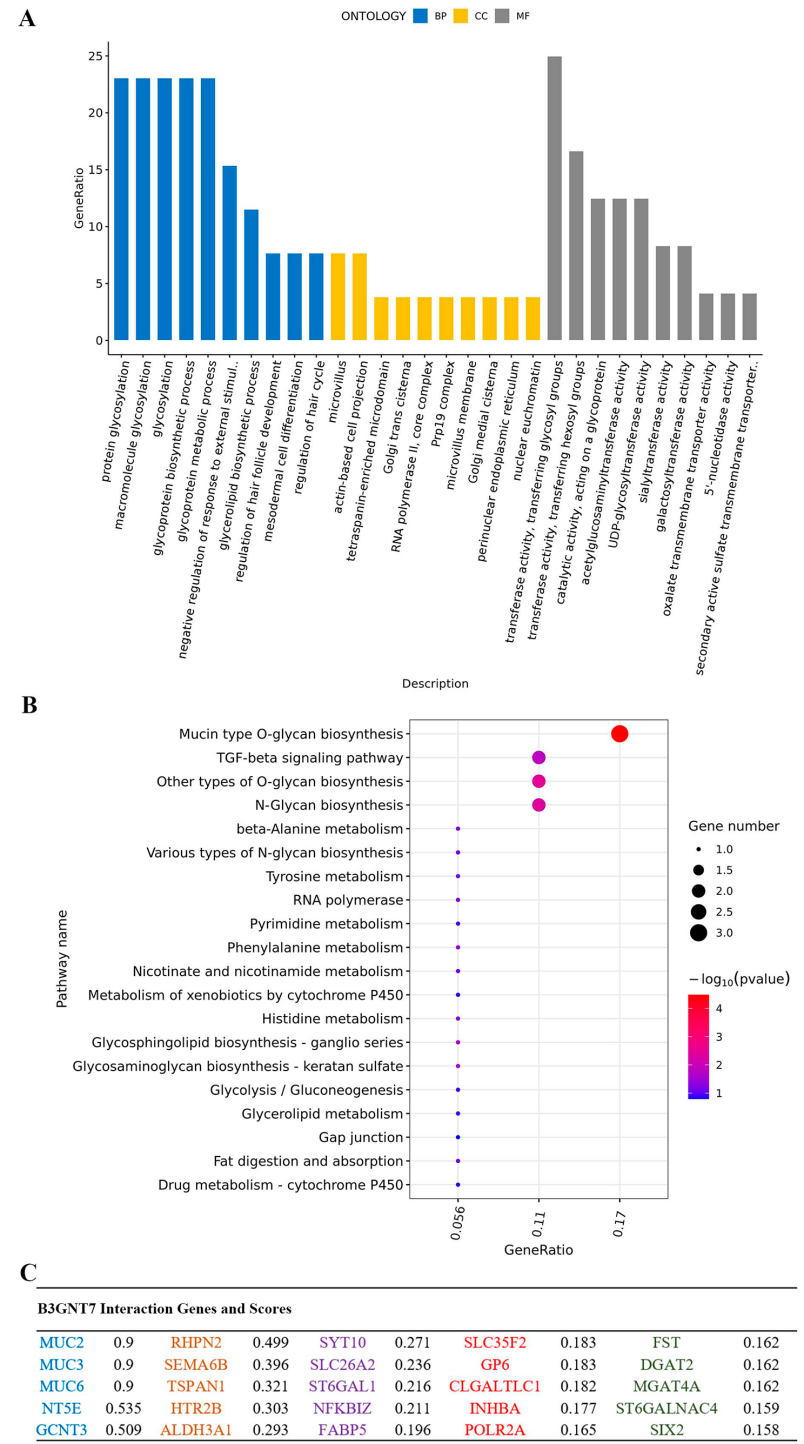
B3GNT7 functional analysis. **A.** Protein interaction analysis using the String database, showing functional enrichment of genes interacting with B3GNT7. GO enrichment analysis revealed involvement in protein glycosylation. **B.** KEGG enrichment analysis identified the Mucin type O-glycan biosynthesis signaling pathway (KEGG: Mucin type O-glycan biosynthesis) as the main pathway involving B3GNT7-interacting genes. **C**. PPI analysis showed that strongly interacting molecules with B3GNT7 include mucin MUC family members, such as Muc2, Muc3, and Muc6

By performing functional enrichment analysis of the B3GNT7, GO enrichment analysis revealed that B3GNT7-interacting genes were mainly involved in the biological function of protein glycosylation. KEGG enrichment analysis showed that B3GNT7-interacting genes were primarily associated with the Mucin type O-glycan biosynthesis signaling pathway (KEGG: Mucin type O-glycan biosynthesis). The construction of the PPI network was of great significance in analyzing signal transduction, gene expression regulation, and functional relationship. According to the rank score, PPI analysis demonstrated stronger interaction between B3GNT7 and the mucin MUC family, including Muc2, Muc3, and Muc6 (Fig. 3).

### B3GNT7 regulates mucin O-glycosylation in patients with UC

**Fig. 4:**
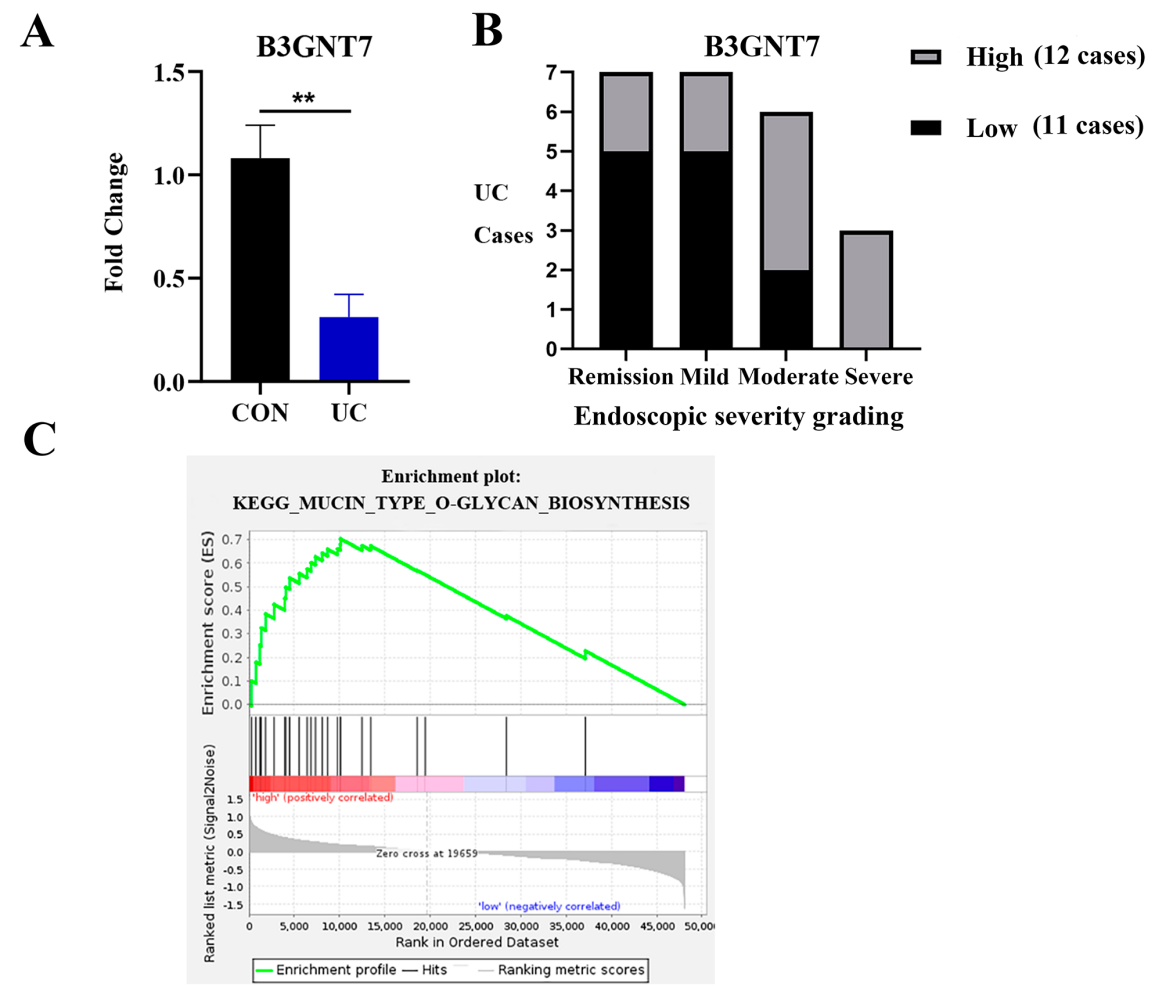
Expression of B3GNT7 in intestinal tissues of patients with UC. **A.** Mining public data from the iHMP on UC patients and healthy controls showed significant down-regulation of B3GNT7 transcriptional level in the UC gut (Fold Change of difference was 0.312, *P* = 0.002 compared to control CON group). **B**. Patients were divided into two groups based on the median of B3GNT7 transcription level: high expression group (*n* = 12) and low expression group (*n* = 11). B3GNT7 expression was higher in the remission and mild active stages of UC and lower in the moderate and severe active stages. The transcriptional level negatively correlated with the endoscopic severity of UC (*P* = 0.025). **C**. Single-gene GSEA enrichment analysis demonstrated significant enrichment of B3GNT7 in the Mucin type O-glycan biosynthesis signaling pathway (KEGG: Mucin type O-glycan biosynthesis) (NES = 1.70, *p* = 0.001)

The Human Microbiome Project (iHMP) IBD database (IBDMDB) was used to select UC cases with complete omics data for this study. The rectal mucosal transcriptome data of 23 UC patients and 20 non-IBD controls were analyzed (Table 1). The results indicated that the transcription level of B3GNT7 in the UC patient tissue was markedly reduced compared to the control group (Fold Change = 0.312, *P* = 0.002). Subsequently, the 23 UC patients were stratified into two groups based on the median B3GNT7 transcript level: the B3GNT7 high expression group (12 cases) and the B3GNT7 low expression group (11 cases). Expression of B3GNT7 was observed to be elevated in the remission and mildly active stages of UC, whereas it was diminished in the moderately and severely active stages. A significant negative correlation was identified between the B3GNT7 transcript level and the endoscopic severity of UC (*P* = 0.025). B3GNT7 was most prominently enriched in the Mucin type O-glycan biosynthesis signaling pathway (KEGG: Mucin type O-glycan biosynthesis) with an NES value of 1.70 and a P value of 0.001 (Fig. 4).

**Table 1:** Brief clinical features of UC patients sourced from the IBDMDB database

Sample number	Gender	Age at diagnosis	Age at the time of sampling	Endoscopic severity grading	B3GNT7 transcription level	B3GNT7 grouping
CSM5FZ1K	Female	29	43	Remission	38,002	High
CSM5FZ1N	Female	33	47	Mild	14,183	High
CSM5FZ1R	Female	58	76	Remission	26,920	High
CSM5FZ1U	Male	24	32	Severe	4425	Low
CSM5FZ2B	Female	25	37	Remission	6517	Low
CSM5FZ2H	Female	13	26	Moderate	1689	Low
CSM5FZ2M	Female	37	50	Severe	15,786	High
CSMDRVXM	Male	20	32	Mild	1376	Low
CSMDRVY1	Female	35	36	Mild	964	Low
CSMDRVY6	Female	28	46	Remission	13,568	High
CSMDTZ4Y	Female	19	42	Mild	14,591	High
ESM5GEYL	Female	17	17	Moderate	9715	Low
HSM5FZBB	Male	13	13	Severe	16,065	High
HSM6S4FS	Female	11	11	Moderate	5021	Low
HSM6S4GI	Male	15	15	Mild	37,758	High
HSM7H3WY	Male	16	16	Severe	3832	Low
HSM9JTC3	Female	13	17	Remission	34,964	High
HSM9UBMQ	Male	16	16	Remission	20,730	High
MSM5LWL1	Female	21	21	Moderate	5152	Low
MSM719MZ	Female	29	29	Mild	2496	Low
PSM6XBZO	Female	16	16	Remission	4087	Low
PSM6XBZQ	Male	16	16	Mild	14,049	High
PSM7J4EW	Male	17	17	Moderate	13,782	High

## Discussion

This study presents the initial documentation of the potential anti-inflammatory role of B3GNT7 in UC. The anti-inflammatory effect of B3GNT7 is hypothesized to be mediated through its involvement in the repair mechanisms of the colonic mucosal barrier, particularly in the context of intestinal mucin glycosylation. Delineating the role of B3GNT7 in colitis facilitates a deeper understanding of the disease’s pathophysiological underpinnings and lays the groundwork for the investigation of novel therapeutic approaches.

B3GNT7 was initially identified in 2002 [9]. The enzyme is tasked with synthesizing glycans and elongating the glycan chain of poly-LacNAc. Despite its discovery, there have been relatively few investigations into B3GNT7, which is notable for its high expression in healthy intestinal epithelial cells [10]. However, its expression is significantly reduced in colon cancer and is inversely correlated with tumor metastasis and prognosis [10, 11]. As expected, B3GNT7 could be used as diagnostic markers of human cancer or target molecules for the development of new therapy [9]. The precise regulatory mechanisms governing B3GNT7 remain elusive. Our study has elucidated a significant role for B3GNT7 in the inflammatory response associated with colitis. Additionally, B3GNT7 is intricately involved in immune regulation within the context of colitis [3]. The IL-22 signaling pathway can activate the transcription of genes crucial for intestinal epithelial cell proliferation, tissue regeneration, tight junction reinforcement, and antimicrobial production [3]. More recent research has hinted at IL-22’s involvement in the modulation of intestinal epithelial fucosylation [12, 13]. In human intestinal epithelial cells, IL-22 signaling alters the expression of the B3GNT7 transcript and stimulates α1-3-fucosylation of glycoproteins, including those presenting mucosal O-linked glycans [3]. Notably, the study identifies the upregulation of B3GNT7 as a pivotal factor in the intensification of fucosylation of O-linked glycans, revealing an unforeseen regulatory mechanism for intestinal fucosylation [3]. Consequently, it is plausible to suggest that dysregulated expression of B3GNT7 may result in immune dysfunctions, triggering or exacerbating the onset of colitis.

Mucins are secreted by goblet cells in the gastrointestinal tract as highly glycosylated proteins, which are rich in o-sugar chains and serve as critical components of the intestinal barrier, safeguarding the gut from pathogenic invasion [14]. Mucins, which are heavily glycosylated in healthy tissues, are overexpressed and exhibit abnormal glycosylation patterns in cancer. MUC1, the first mucin to be structurally elucidated, has been increasingly recognized for its role in protecting against infection as part of the body’s mucosal barrier. The interaction between microbes and MUC1 offers a glimpse into the biochemical modifications of the proteins involved, which could serve as targets to inhibit the development of cancer. The glycosylation state of MUC1 is a key determinant of its function, with alterations in glycosylation allowing the same mucin to behave differently in cancerous versus normal cells [15]. MUC2, a pivotal constituent of the intestinal mucus layer, adheres to intestinal epithelial cells to preserve intestinal mucosal homeostasis [16, 17]. Mucin O-glycans are the predominant glycans in the gut, and mutations in genes related to their synthesis can result in abnormal glycosylation of intestinal epithelial cells, which is a significant factor in the onset of colonic inflammation [18]. The disruption of intestinal glycan balance, in conjunction with gut microbiota and mucosal immunity, is a contributing factor to the development of IBD [19]. Research has revealed that mice with deficiencies in intestinal glycan exhibit a depletion of colonic mucosa-associated intestinal microbiota, leading to spontaneous colitis [20]. Impairments in glycosyltransferase-mediated glycan synthesis can lead to abnormal glycosylation patterns in the gut. O-glycosylation of mucin MUC2 is implicated in colonic inflammation in UC. O-glycosylation modifications of intestinal epithelial cells are significantly reduced in patients with IBD [21]. The aberrant expression and glycosylation modifications of the mucin family in the context of colitis suggest that B3GNT7 may interact with mucins secreted by goblet cells and contribute to the restoration of intestinal barrier function. In future research, a cell-based mucin tandem repeat array platform can be used as a valid model to study O-glycosylation of mucins [22].

Further in-depth exploration is warranted to delve into the protective potential of B3GNT7 against colitis, as indicated by our findings. Several areas of investigation can be pursued. First, a comprehensive study of the functional mechanisms of B3GNT7 is necessary, including its influence on the glycosylation of adhesive molecules, such as mucins, and its regulatory role in pertinent signaling pathways. Second, the interactions between B3GNT7 and other pivotal molecules, such as T cell receptors and inflammatory factors, should be examined to elucidate B3GNT7’s specific role in immune modulation. Third, experimental studies utilizing animal models and clinical specimens are essential to corroborate the functions and mechanisms of B3GNT7, thereby further affirming its significant role in colitis. Finally, the exploration of therapeutic interventions aimed at modulating B3GNT7, such as its activation or inhibition, could potentially ameliorate the pathological progression of colitis. One limitation of our study is that there is no validation using cell lines or mouse models, and we have not further investigated the interaction between target genes/proteins by conditionally knocking out B3GNT7 animal models and cell culture. In future research, this is worth further in-depth investigation.

## Conclusion

Our study found that the downregulation of B3GNT7 expression in the colonic tissues of UC patients may contribute to the compromised mucin barrier function and the exacerbation of colitis. The potential anti-inflammatory role of B3GNT7 in UC, likely through its involvement in colonic mucosal barrier repair, particularly in intestinal mucin glycosylation. This finding deepens our understanding of UC’s pathophysiology and opens avenues for novel therapeutic approaches.

## Data Availability

The RNA-seq data of mice was deposited in the GEO database (http://www.ncbi.nlm.nih.gov/geo/) under the accession number GSE244377. The data of UC patients used to support the findings of this study was downloaded from the IBDMDB database (https://ibdmdb.org/) with GEO series accession number GSE111889.

## Abbreviations

UC: Ulcerative colitis
IBD: Inflammatory bowel disease
GSEA: Gene set enrichment analysis
DSS: Dextran sulfate sodium
DEGs: Differentially expressed genes
GO: Gene ontology
KEGG: Kyoto encyclopedia of genes and genomes
GEO: Gene expression omnibus
PPI: Protein-protein interaction
iHMP: The integrative human microbiome project
CON: Control group
MUC2: Mucin 2
IHC: Immunohistochemistry
